# Reverse Engineering of Bacterial Chemotaxis Pathway via Frequency Domain Analysis

**DOI:** 10.1371/journal.pone.0009182

**Published:** 2010-03-09

**Authors:** Junjie Luo, Jun Wang, Ting Martin Ma, Zhirong Sun

**Affiliations:** 1 Ministry of Education Key Laboratory of Bioinformatics, Department of Biological Sciences and Biotechnology, Tsinghua University, Beijing, People's Republic of China; 2 Department of Computer Science, Tsinghua University, Beijing, People's Republic of China; Virginia Tech, United States of America

## Abstract

Chemotaxis is defined as a behavior involving organisms sensing attractants or repellents and leading towards or away from them. Therefore, it is possible to reengineer chemotaxis network to control the movement of bacteria to our advantage. Understanding the design principles of chemotaxis pathway is a prerequisite and an important topic in synthetic biology. Here, we provide guidelines for chemotaxis pathway design by employing control theory and reverse engineering concept on pathway dynamic design. We first analyzed the mathematical models for two most important kinds of *E. coli* chemotaxis pathway—adaptive and non-adaptive pathways, and concluded that the control units of the pathway *de facto* function as a band-pass filter and a low-pass filter, respectively, by abstracting the frequency response properties of the pathways. The advantage of the band-pass filter is established, and we demonstrate how to tune the three key parameters of it—A (max amplification), ω_1_ (down cut-off frequency) and ω_2_ (up cut-off frequency) to optimize the chemotactic effect. Finally, we hypothesized a similar but simpler version of the dynamic pathway model based on the principles discovered and show that it leads to similar properties with native *E. coli* chemotactic behaviors. Our study provides an example of simulating and designing biological dynamics *in silico* and indicates how to make use of the native pathway's features in this process. Furthermore, the characteristics we discovered and tested through reverse engineering may help to understand the design principles of the pathway and promote the design of artificial chemotaxis pathways.

## Introduction

The ability of motile microorganisms to respond to chemical gradients in their environment and direct their movement accordingly is defined as chemotaxis [1], [2]. One typical bacterial chemotactic behavior is the chemotaxis of *Escherichia coli*, whose molecular mechanism has been thoroughly investigated in the last three decades [2]–[5]. Flagellum rotation, key to *E. coli's* mobility, is driven by a molecular motor switching between two operational modes. Counter-clockwise (CCW) gyration of the motor entangles flagella into a bundle driving the bacteria towards a fixed direction, while clockwise (CW) gyration corresponds to the tumble and reorientation of the bacteria. The motor rotation bias (the fraction of time the motor spend on certain state [6], [7]) is controlled by the phosphorylation state of a motor-binding protein called CheY, whose phosphorylation level is positively related to the CW rotation bias [8]–[10]. Specific receptors, which locate in the very beginning of the chemotaxis pathway, involve in the sensing of attractantsand transduce the signal via the pathway to the motor to control its rotation mode. Investigators attempt to discover essential properties which enable this pathway to direct chemotactic behaviors by establishing and analyzing its mathematical model [11]–[13]. Various features, such as amplification rate, adaptation robustness, relaxation time and feedback loop, are highlighted in their researches. However, in the scope of synthetic biology, these fragmented features are necessary rather than sufficient for pathway design. So far researchers merely prove the necessity of these features in realizing chemotaxis, while the sufficiency is still open to doubt.

**Figure 1:**
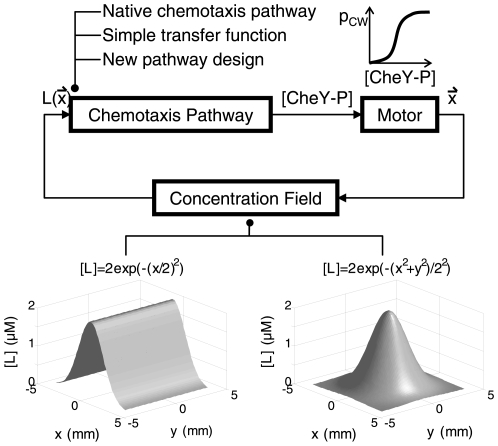
System block diagram of the chemotaxis model. Chemotaxis pathway senses the ligand concentration and outputs the signal controlling motor bias in the form of [CheY-P]. Molecular motors on the cell surface can change cell's position or direction. In our research, the reverse engineering of the pathway is divided into three steps: analyzing the dynamic properties of native chemotaxis pathway; selecting and optimizing the transfer function describing the pathway dynamic; designing new pathway based on the transfer function. We changed the situation in our simulation by modifying the concentration field module in our program. The ligand concentration fields used here are mountain-shaped or peak-shaped.

Furthermore, we hypothesized a similar but simpler version of the chemotaxis dynamic pathway model by assembling the essential features of the native pathway together. From the view of control theory, chemotaxis pathway corresponds to the controller in bacterial moving system (Fig. 1). A widely-used design approach for controller is to devise its transfer function basing on the transfer function of the effecter (in this case, molecular motor). However, the motor's working manner is discrete and stochastic, thus it is impossible to formulate it by transfer function. Therefore, the above-mentioned method cannot be used in our project. Here we circumvent this problem by adapting reverse-engineering methodology. We abstracted the kinetic relationship between the input (ligand concentration [L]) and output (the concentration of phosphorylated CheY [CheY-P]) in wild type chemotaxis pathway, and wrote controller's transfer function accordingly. Then, we assembled the essential features of the native pathway revealed in our study into a new pathway, using on the optimized transfer function. Subsequent evaluation by computational simulation proves its sufficiency to function well. For the reason that the design is based on reverse engineering, our findings are also helpful to understand the design principles of native chemotaxis pathway. Furthermore, our work enriches mathematical methods available for synthetic biology.

## Results

### Frequency-Domain Analysis of Typical Chemotaxis Pathway

In order to understand how chemotaxis pathway leads *E. coli* to find its favorite environment, we employed frequency-domain analysis in control theory to extract its key features. As mentioned, chemotaxis pathway corresponds to the controller of *E. coli* movement system in terms of control theory (Fig. 1). The strategies for chemotaxis pathway construction can be classified into two types according to whether they are adaptive. Typical native chemotaxis pathway is perfectly adaptive to many ligands, such as aspartate [7], [12], [14], [15] while a minority of native pathways (for example of which detecting O_2_ [16]) as well as pseudochemotaxis pathways (artificial ones designed to sense certain small molecule [17]) are not adaptive. These two pathway models have been fully investigated, in that their mathematical models have been established. Here we linearized those pathway models near equilibrium to get their respective approximate transfer functions and perform frequency-domain analysis. The two pathway models analyzed here were previously described by Goldstein et al. [18] and Emonet et al. [11]. Goldstein et al. proved the advantages of non-adaptive dynamics, which means [CheY-P] directly relates to stimuli, by carrying *in silico* chemotaxis evolution. While the mathematical model of Emonet et al. focuses on aspartate chemotaxis pathway, a typical adaptive pathway.

**Figure 2:**
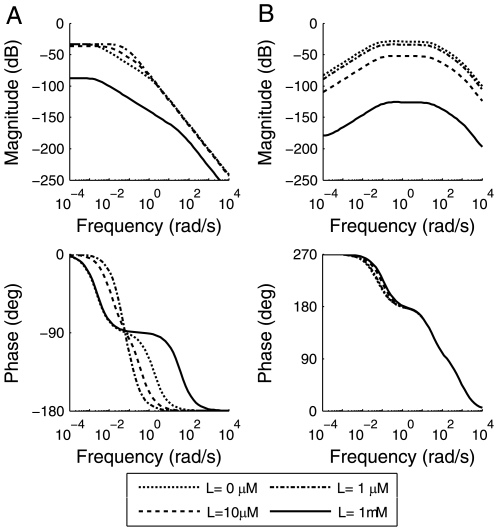
Bode diagrams of non-adaptive and adaptive chemotaxis pathway. The figure shows the magnitudes and the phase shifts (with respect to the sinusoidal [L] signal) of [CheY-P] against different signal frequencies in non-adaptive chemotaxis pathway (A) and adaptive chemotaxis pathway (B) near typical steady states under four ligand concentrations: 0 µM,1 µM, 10 µM and 1mM, which can be further categorized into three working conditions (quiet condition: 0 µM; a fraction of receptors are occupied:1 µM and 10 µM; all receptors are saturated:1mM).

Bode diagrams in Fig. 2 show frequency responses of these two pathways near typical steady states under four [L]s: 0 µM,1 µM, 10 µM and 1mM, which can be further categorized into three working conditions (quiet condition: 0 µM; a fraction of receptors are occupied:1 µM and 10 µM; all receptors are saturated:1mM). The differences between the curves of these working conditions are due to the nonlinear effects of the pathways. As expected, the magnitude of pathway is much lower in the saturation condition than others. It is evident from Fig. 2A that the non-adaptive pathway here is a low-pass filter allowing sine signals whose frequencies are lower than a certain cut-off to pass while attenuating signals above the threshold. This means the pathway is sensitive to the [L] changing relatively slowly but not that fast fluctuating. Besides, adaptive pathway functions as a band-pass filter. It has two cut-off frequencies and only [L] signals with frequencies in between filter through (Fig. 2B). Eliminating low frequency signals means constant stimuli, whose frequency can be considered as 0, is not able to trigger the pathway. In fact, it can be proved that the 0 magnitude to 0 Hz signal is equivalent to the perfectly adaptive pathway (see Supplemental Material Text S1). Consequently, band-pass property leads to adaptation. As these two kinds of pathway both exist in nature, and were previously shown to bear their own merits [14], [18]–[21], we first need to characterize and compare the two design strategies in detail.

### Test the Chemotaxis Effects of Low-Pass and Band-Pass Filter

In order to test the chemotaxis effects of above two design strategies, we here apply two simple transfer functions to describe the dynamic relationships between receptor occupancy and [CheY-P] variance from a base line. Then we simulated the movement of bacteria under the control of these two types of filters. The base line of [CheY-P] is set to 2.71 µM, corresponding to 20% CW rotation bias. This makes the motility of unstimulated bacteria consistent to experiment [11], [22], [23]. Two most important nonlinear processes, which are not possible to be incorporated into the transfer function by approximation, exist in this pathway: 1) the binding of ligand to receptors, in which a very high ligand level may cause receptor saturation; and 2) CheY-P controlled motor state, which is extremely discrete and stochastic. This is the reason why we only use transfer function to describe the dynamic relation between receptor occupancy and [CheY-P]. On the other hand, even in extreme cases when this part deviates much from equilibrium in a way that linearization will cause substantial error in [CheY-P], the output ([CheY-P]) will fall into the saturated or dead zone of the motor in which motor response is all or none, so the final result will still make sense. The two transfer function corresponding to low-pass filter and band-pass filter are as following:
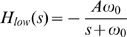
(1)

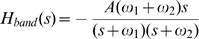
(2)


The influences of max amplification (A) and cut-off frequencies (ω_0_, ω_1_, ω_2_) to chemotactic effect are tested in our simulation. The range of the cut-off frequencies used in the parameter scan is wide enough to cover both fast (e.g. phosphorylation) and slow (e.g. transcriptional regulation) biological processes. We simulated the movement of bacteria on petri disks, and found that the distribution of the bacteria is similar to the actual pattern obtained in experiments (simulated videos are available at http://www.bioinfo.tsinghua.edu.cn/luojunjie/chemotaxis_movie/), which offers a strong evidence that our approximation is reasonable.

### Chemotactic Effect of Low-Pass Filter

Obviously, the chemotaxis effects of the low-pass filter is determined by two parameters in the transfer function, A and ω_0_, which correspond to the amplification and delay of the pathway, respectively. First, the max amplification A is positively related to the chemotaxis effects, if chemotaxis behavior is detected. A high amplification of the controller indicates that the pathway is sensitive to stimulation. The sensitivity should be strong enough to offset the tendency of stochastic movement, or otherwise, bacteria population would merely randomly swim in the median (Fig. S1). Second, the cut-off frequency ω_0_ should be high enough to enable the pathway to response to fast-changing ligand concentration. This transfer function is a typical inertia link. A low ω_0_ causes bacteria to respond to the input with a long delay. In this case if a bacterium is running in a wrong direction, it takes much time for the controller to transduce the signal to the motor and correct the cell; similarly if the direction of the bacterium movement happens to be correct, CCW bias of the motor cannot increase in time to keep the running state. Thus only the controller with very high max amplification and cut-off frequency shows significant chemotaxis behaviors (Fig. S1I).

One important feature inherent to this transfer function is non-adaptation, which means the steady state output of the pathway is related to the input signal level. Even though its amplification and cut-off frequency are both high enough, it still has some disadvantages. First, the mobility of the cell is high where [L] is high, which disables *E. coli* from maintaining their position where optimal [L] is achieved by favoring both approaching and departing. Therefore, the cell population spread in a wide area around the target. The population average ligand concentration oscillates (Fig. S1I), indicating that bacteria run over the concentration peak but do not turn back within a short distance. Second, once a bacterium enters a low concentration zone, its motility decreases so dramatically that it keeps tumbling there (Fig. S1G–S1I).

### Chemotactic Effect of Band-Pass Filter

**Figure 3:**
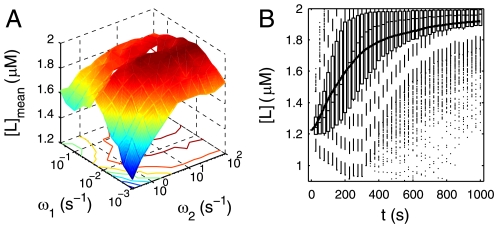
Chemotactic effects of the band-pass filter. (A) Effects of variations of ω_1_, ω_2_ on the average ligand concentration. The transfer function of the pathway is as shown in Eq. 2. The max amplification of the filter is fixed at 16. Each data point represent the movement of 100 bacteria in a mountain-shape concentration gradient (L = L_0_exp(−x^2^/r^2^), L_0_ = 2 µM, r = 2 mm). The initial position of all the bacteria is (1.4 mm, 0 mm). 1000 s running was simulated and the average [L] in the last 50 s are calculated as a measurement of chemotactic effects. (B) Chemotaxis behaviors of cells guided by a band-pass filter with ω_1_ = 0.02 s^−1^, ω_2_ = 5 s^−1^ in a mountain-shaped concentration field. The concentration field and initial point is the same as that in (A). Here, 1000 bacteria are simulated and their local [L] distribution is shown by box plots at each time point. Each box has three lines, which from low to high indicate the lower quartile, median, and upper quartile of [L] at a given time. Whiskers extend from the box out to the most extreme data value within 1.5 folds of the height of the box. [L] values beyond whiskers are marked by points. Solid line shows the average of [L] against time. The average ligand concentration increases fast and achieves a high final value (>1.9 µM) without oscillation. Few bacteria are trapped in places with low ligand concentration.

First of all, we investigated how the three parameters in the transfer function (A, ω_1_ and ω_2_) influence the chemotaxis effects. We simulated the movement of 100 bacteria and plotted the average [L] around each cell from 950 s to 1000 s to describe the chemotactic effect (Fig. 3A). The reason why we average this value in last 50 s is to reduce fluctuation resulted from the movement randomness. This criterion can integrate the speed the average ligand concentration become stable and how large the final stable value bacteria can achieve (Fig. S2). Similar to the low-pass filter, magnitude contributes to the chemotaxis effect (Fig. 3A, S3A and S3B). A good chemotaxis effects can be much more easily achieved by this band-pass filter comparing to the low-pass one. The max amplification of the pathway in Fig. 3B is only 16, but it can lead to a higher final average [L] (>1.9 µM, Fig. 3B) than that non-adaptive pathway whose max amplification is 64 does (about 1.6 µM, Fig. S1I).

Fig. 3A shows the average [L] after 1000 s running under different ω_1_, ω_2_. Generally, to a fixed down cut-off frequency ω_1_, as up cut-off frequency ω_2_ increases from 0.3 s^−1^ to 100 s^−1^, the final average concentration increases. But this contribution is not noticeable once ω_2_ is higher than 5 s^−1^, resulting from the minimum response time limitation of the motor, which is set at 0.5 s in our model. Signals whose periods are much shorter than it can hardly influence the working state of the molecular motor.

Interestingly, the down cut-off frequency plays a critical role in adaptation via regulating the ‘memory’ of this pathway. Its best value is about 0.01 Hz or 0.02 Hz, which strikes a balance between sensing concentration ramps and adapting to long term stimulation, resulting in prompt approaching the concentration peak and maintaining its position (Fig. S2D–S2F). We can see from our simulation that if ω_1_ is too high, the ability of keeping the right direction is abolished, so the population average [L] increases relatively slow (Fig. S2A–S2C); and if ω_1_ is too low, when a bacterium runs over the concentration peak, it still “remembers” the former concentration rise and is misinformed to be swimming in the right direction so that cannot brake in time (Fig. S2G–S2I).

The controller described by Eq. 2 is perfectly adaptive, because its numerator is a differential link, which means calculating the differential of receptor occupancy. The sign of the outcome of the link indicates whether a bacterium is running up or down the concentration gradient. Compared to low-pass filter, the adaptation property of band-pass filter makes cell motility at steady state independent of input [L]. Thus, the distribution of bacteria around position with the highest [L] is more converged, and they would not be trapped at places with very low [L] (Fig. 3B).

We also investigated chemotaxis behavior controlled by band-pass filter in different conditions. The conditions we tested include placing bacteria at a position farther from the max ligand concentration (Fig. S3C), sharper concentration gradient (Fig. S3D) and a peak-shaped concentration field (Fig. S3E, S3F). All the parameters of each subfigure in Figure S3 are listed in Table S1. We also tested band-pass filters with ranks higher than Eq. 2, like the one shown in Eq. 3. (4 rank), which has the same max amplification and cut-off frequencies. The chemotactic effects of it are shown in Fig. S3G, S3H.
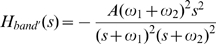
(3)


It is interesting that all these simulations show very similar influences of magnitude and the two cut-off frequencies to chemotaxis effect (including higher rank band pass filters, provided the up and down cut-off frequencies are the same): high magnitude and up cut-off frequencies improve chemotactic result and the best down cut-off frequency is about 0.01 Hz or 0.02 Hz. Our finding here is possible to be an important guideline for chemotaxis pathway design. Better chemotactic ability, which could be recapitulated by the above-mentioned parameters, confers evolution advantage. Consequently, examining whether relevant properties of wild type *E. coli* are consistent with the best parameter we find is a touchstone to check the reliability of our work. If bacteria are stimulated by step-wise addition or removal of chemotactic ligand, the phosphorylation proportion of CheY increases dramatically but gradually returns to the basal level. The time half of recovery takes is called adaptation time, which is mainly determined by the down cut-off frequency. The best adaptation time deduced from our simulation is about 40s (Fig. S4), which is consistent with the study done by Sourjik et al. [24] who measured CheY phosphorylation after step-wise stimuli.

### Design Biochemical Dynamics of New Chemotaxis Pathway Based on Transfer Function and Test Its Chemotactic Effects

**Figure 4:**
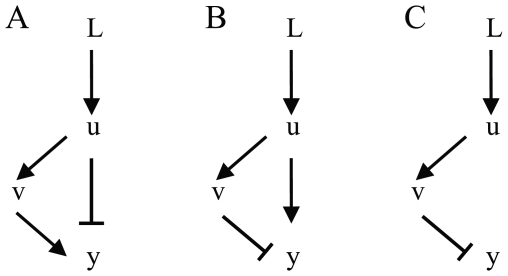
Designed dynamics of positive, negative and pseudo- chemotaxis pathway. (A) Positive chemotaxis pathway. (B) Negative chemotaxis pathway. (C) Pseudochemotaxis pathway.

Based on the study on the kinetic features of the native chemotaxis pathway, we were able to design a novel pathway to control bacteria tropism by reversing the process from biological dynamics to transfer function. Traveling up the attractant concentration gradient is defined as positive chemotaxis, while the swimming away from repellants is called negative chemotaxis. We designed a pathway containing three molecules to realize a band-pass filter for positive chemotaxis. This pathway is proved to be the smallest structure that works as a band-pass filter (see Text S1). Their dynamic interactions are shown in Fig. 4A: ligand quickly binds to molecule u to activate it. Activated u can dephosphorylate CheY-P and generate active molecule v. Active molecule v phosphorylates CheY. So when this pathway is stimulated by chemical attractant, the concentration of CheY-P decreases at the beginning, but as active molecule v accumulates in the cell, the dephosphorylation effect of u is neutralized, so the pathway adapt to the new ligand concentration (Fig. 4A). Similarly, if we swap the phosphorylation and dephosphorylation effect of u and v, a negative chemotaxis pathway can be generated (Fig. 4B). The dynamic of the designed positive chemotaxis pathway can be described by following differential equations:
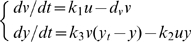
(4)The equilibrium value of y and the transfer function of the pathway can be derived from Eq. 4:
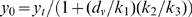
(5)

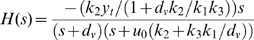
(6)


**Figure 5:**
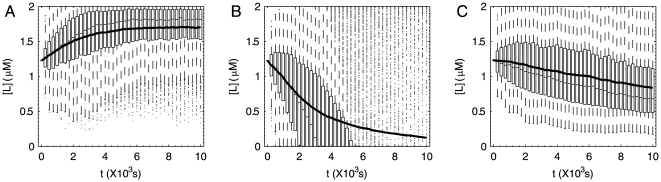
Chemotaxis results of positive, negative and pseudo- chemotaxis. Movement of 1000 bacteria is simulated in a mountain-shaped concentration field as the same in Fig. 3(A). Box plots show the distribution of their local [L] at each time point. Solid line shows the average of [L] against time. Both positive (A) and negative (B) chemotaxis pathways we designed effectively exhibit chemotaxis behaviors, but the chemotaxis behavior of pseudochemotaxis pathway (C) is not significant comparing to them.

**Figure 6:**
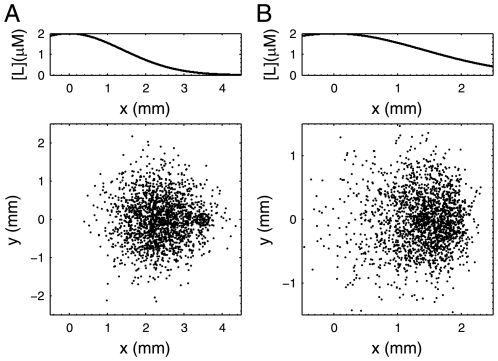
Simulated distribution pattern of cells placed in attractant gradient (L = L_0_exp(−x^2^/r^2^), L_0_ = 2 µM, r = 2 mm). The curve of the [L] against x is shown. (A) Simulated distribution of 2000 bacteria guided by the positive chemotaxis pathway. The initial position of the bacteria is (3.5 mm, 0 mm). (B) Simulated distribution of 2000 bacteria guided by the pseudochemotaxis pathway. The initial position of the bacteria is (1.4 mm, 0 mm). These two distribution patterns are very similar to actual experiment result [17].

Evidently, the form of transfer function is made up of one differential link and two inertial links, very similar to Eq. 2. But because of nonlinear properties of biological systems, the magnitude and cut-off frequencies of the controller may vary under different [L] input. We keep the most important feature, down cut-off frequency d_v_ (molecule v deactivation rate) at the optimal value 0.02 s^−1^, the magnitude (pathway sensitivity)as high as possible and up cut-off frequency (∼CheY-P dephosphorylation rate) high enough. In order to exclude any error stemming from the linear approximation, we tested the control abilities of the pathway designs and observed the chemotactic behaviors by our program based on the original differential equations (Fig. 5). For comparison, a non-adaptive pathway derived from the above two pathways was simulated together as a control. As expected, the positive and negative chemotaxis pathways are able to lead to significant average [L] increase and decrease, although not as good as that in Fig. 3B because of limited amplification. The distribution of bacteria guided by positive designed chemotaxis pathway is nearly the same as the wild type cells. Both of them travel to the attractant and form fan-shaped patterns on the plates (Fig. 6A). In contrary, when the distribution of bacteria is guided by the designed non-adaptive pathway (pseudochemotaxis), it tends to defuse faster where the attractant concentration is higher, as shown in Fig. 6B. In this case, the cells are hard to stay at the position with high ligand concentration and easy to be trapped where ligand concentration is low, which accounts for the slight decline of the average [L] in Fig. 5C. The consistency between the simulation of designed pathways and actual experiment results [17] proves both the reliability of the simulation platform and the validity of our pathway design.

## Discussion

### Nonlinear Effects Lead to Robustness of Perfect Adaptation in the Designed Pathway

Some investigators consider the integral feedback as the key structure for robust perfect adaptation, which means if the activity of some molecules in the native chemotaxis pathway is changed, the pathway is still perfectly adaptive [14], [19]. However, though the pathway we designed is absence of any feedback loops, it is still perfectly adaptive because of its nonlinear properties. We call this pathway structure “asymmetric clamp”. Input signals are transmitted to CheY-P through the long and short arms of this “clamp”. Both of them are low-pass filters but have different cut-off frequencies. Signals in low frequency regime going through the two filters neutralize at CheY-P (Fig. S5), ensuring the overall pathway functions as a band-pass filter. At a first glance, the two arms' gains need to be fine tuned to counterbalance each other. But it is interesting that the amplifications of the two arms are always the same under different input levels and parameter values, because the activation and deactivation speed of CheY are always in proportion at steady state. Therefore, the perfect adaptation of pathway design is robust. The steady state value of output is independent of input (Eq. 5) can be considered as criteria for robust perfect adaptation. It is equivalent to the another criteria that H(0) = 0 (Eq. 6) (see Text S1).

### Apply Control Theory in Synthetic Biology

Our work provides an example of applying control theory in dynamic model design (an important topic in synthetic biology). Bacterial chemotaxis pathway design mainly involves two aspects: one is the sensing of attractants or repellents by macromolecules and relaying these signals to the motor; the other is the quantitative relations between the input ligand concentration and output signal controlling motor rotation state. The second one is usually overlooked in previous studies, resulting in the previous pale imitation of the native chemotaxis process. Control theory is a powerful tool for designing system dynamics. The method used in our study, that is deriving transfer function from the mathematic model of a pathway, bridges the two realms. Linearizing the model describing a biological system is an approximate, which is only applicable to biological systems whose nonlinear effects are not very strong, but not to those working in saturation state or dead zone.

Transfer function and bode diagram are useful tools to describe the dynamics of a pathway in two aspects. First, they distill several “key parameters”, which are tightly related to the dynamic properties of the pathway, like A, ω_0_, ω_1_ and ω_2_ in our research from large numbers of parameters in the pathway. This makes it much easier to scan the parameter space to determine the optimal value. And more importantly, different dynamic properties can be changed independently in this scanning. For instance, if we try to solely regulate the relaxation time of the pathway by changing ω_1_, amplification or moving tendency of the cells will remain constant. Second, many properties of the bacterial chemotaxis are reflected in the transfer function. For example, by analyzing the transfer function, our research sheds light on the question of how *E. coli*, which is only capable of sensing its local chemical concentration, senses the concentration gradient in the environment to determine its moving direction. We reason that the differential link in Eq. 2, which means calculating the derivation of [L], indicates whether or not the bacterium is running in a right direction.

### Reverse Engineering in Pathway Design

Reverse engineering is the process of discovering the technological principles of a device, object or system through analysis of its structure, function and operation by taking it apart, analyzing its workings in detail, and manufacturing a new one with similar function to the original [25]–[27]. Our work demonstrates that reverse engineering is helpful to learn the design principles form the native chemotaxis pathway and design a new pathway model by circumventing the problem that the working manner of a motor cannot be described by transfer function.

We need to know the transfer function of a biological controller in order to carry on reverse engineering. There are several approaches to uncover the frequency domain characteristics of the pathway and then derive its approximate transfer function. The one used here is calculating from the mathematical model based on detailed biochemical mechanisms, which relies on the existing knowledge of detailed pathway mechanisms. The second approach is to consider the controller need to be mimicked as a black box. We can input sine signals with different frequencies into it and analyze the magnitudes and phase shift of the output [6], [7], [27], or measure the fluctuation of input and output, and deriving the frequency-domain characteristics from power spectrum [11], [23], [28], [29]. This approach does not ask for the mechanism of the controller, but needs complex equipment and sensitive probes to create input signals and detect output signals. In our approach, we draw lessons from the native chemotaxis pathway by reverse engineering it to design and optimize a simplified pathway without knowledge of detailed. The designed pathway has special dynamics to be functional for biological systems to work and it may show how to be enlightened by corresponding structure in nature.

## Methods

### Transfer Function of Pathway Near Equilibrium

The differential equation model for a signaling pathway can be written as:
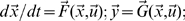
(7)

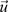
 is the pathway's input, which can be the concentration of ligand ([L]) or activated receptor. 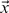 is concentration of molecular components in this pathway. 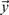 is pathway's output, standing for the concentration of CheY-P ([CheY-P]) in our research. We linearize above differential equations around equilibrium via one order Taylor expansion and ignore infinitesimal of higher order:
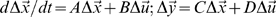
(8)In these equations, *A_ij_ = ∂F_i_/∂x_j_*, *B_ij_ = ∂F_i_/∂u_j_*, *C_ij_ = ∂G_i_/∂x_j_*, *D_ij_ = ∂G_i_/∂u_j_*; 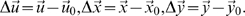 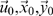 are the values of 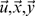 at equilibrium, which means the state satisfies 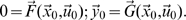
Transfer function describing the dynamic relationship between input and output is derived from Eq. 8:
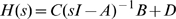
(9)


### Frequency Domain Analysis

The steady-state response of a system to sinusoidal input signal is defined as frequency-domain response. A signal can be decomposed into a set of sinusoidal signals by Fourier decomposition, thus the frequency domain properties reflect the dynamics of a system. If the system is linear, sinusoidal inputs always lead to sinusoidal outputs with the same frequency. The correlation between input and output can be characterized by two parameters, namely the Magnitude (the proportion by which the system amplifies the input sine wave) and the phase shift (the degree by which the output sine wave is delayed comparing to the input). To a linear system whose transfer function is H(s), its magnitude and phase shift to input with an angle frequency ω corresponds to the modulus and phase angle of the complex H(ωj) [30].

Bode diagram shows the magnitude and phase shift of the frequency response of a linear system under different frequency. The magnitude is plotted in decibels (dB, computed as 20log_10_|H(ωj)|), and the phase in degrees.

### Modeling Movement of Bacteria

Our simulation program is made up of three modules: concentration field, motor and pathway (Fig. 1). Concentration field module describes the concentration of target chemical in each location. We simulate the movement of bacteria on plate, so the positions of *E. coli* are described by 2 dimensional vectors. The shape of the field can be set in each simulation. In most of the experiments, we use mountain-shaped field ([L] = L_0_exp(−(x/r)^2^)). In other cases, a peak-shaped ([L] = L_0_exp(−(x^2^+y^2^)/r^2^)) field is employed. We apply a Markov chain with states 0 (tumble) and 1 (run) to represent the working state of molecular motor impelling *E. coli*. The state of molecular motor is approximated constant in a short time scale (0.5 s here, which is selected according to the research done by Ishihara et al. [31]). The state in the next time scale is determined by current state and [CheY-P]. The correlation between [CheY-P] and transition probability matrix of the Markov chain is calculated and checked basing on the research completed by Cluzel et al. in 2000 [32] (see Text S1). Pathway module in our program is a set of standard differential equations expressing the dynamic relationship between [L] and [CheY-P] (Eq. 7).

In each simulation, all the bacteria start at the same initiation position but their orientations are randomly selected. The initial concentrations of molecules in the pathway are set at equilibrium. In each time scale, if the motor state is 1, the cell runs forward at a speed 0.02 mm/s [33]; otherwise, it tumbles and reorients, and distribution of direction deflection follows the finding of Liu et al [33]. At the end of each time scale, the [CheY-P] is calculated and this value influences motor state in the next time scale. This cycle is repeated to see notable chemotactic behavior. We also derive a partial differential equation group to describing the evolvement of the bacteria distribution (see Text S1). This is the first equation group combining the distribution of bacteria and molecular mechanism of chemotaxis, and it is equivalent to the above simulation.

### Systematic Design a Pathway Basing on Defined Transfer Function

To design the pathway systematically, we use a universal pathway model similar to the one described by Soyer O.S. et al [18], [21]. This model assumes that a pathway consist of n molecules, all of which can switch between deactivated or activated state. The active form of each molecule is able to activate or deactivate of other molecules. The first molecule u works as a receptor whose activity is determined by [L] (receptor occupancy u = [L]/([L]+K_L_)), while last molecule is CheY and the concentration of its active form influences motor rotation bias. The biochemical dynamics for all concentration of activated molecules are described by following equations:
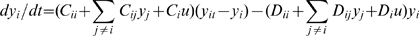
(10)Where y_it_ is the total concentration of other molecules in the pathway except the receptor, and y_i_ is the concentration of activated molecule i. C_ij_ (D_ij_) is the rate at which activated molecule j activates (deactivates) molecule i, C_ii_ (D_ii_) is molecule i's self-activation (deactivation) rate, C_i_ (D_i_) represents the rate at which the receptor activates (deactivates) the molecule i. Transfer function describing the correlation between u and [CheY-P] can be derived through the method described above (see Text S1). This model enables us to find out which pathway topology can generate the transfer function we need, and how to regulate the parameters in the pathway to achieve the design targets.

## Supporting Information

Text S1 Supplemental materials. Exposition and derivation of transfer function of pathway near equilibrium and the model for molecular motor in *E. coli*.
(0.52 MB DOC)

Figure S1 Chemotaxis behaviors of cells guided by low-pass filter with various parameters (ω_0_, A, whose values are shown above each subfigure) in a mountain-shaped concentration field. The concentration field is mountain-shaped (L = L_0_exp(−x^2^/r^2^), L_0_ = 2 µM, r = 2 mm). In each subfigure, 100 bacteria start at (1.4 mm, 0 mm) and their movement in 1000s is recorded. The distribution of their local ligand concentrations is shown by box plots at each time point. Each box has three lines, which from low to high indicate the lower quartile, median, and upper quartile ligand concentration values ([L]) of bacterium population at a given time. Whiskers extend from the box out to the most extreme data value within 1.5 folds of the height of the box. [L] values beyond whiskers are marked by points. Solid line shows the average of [L] against time.
(2.11 MB TIF)

Figure S2 Chemotaxis behaviors of cells guided by band-pass filter with various cut-off frequencies (ω_1_, ω_2_, whose values are shown above each subfigure) in a mountain-shaped concentration field. The max amplification of the filter is fixed at 16. The concentration field is mountain-shaped (L = L_0_exp(−x^2^/r^2^), L_0_ = 2 µM, r = 2 mm). In each subfigure, 100 bacteria start at (1.4 mm, 0 mm) and their movement in 1000s is recorded. The distribution of their local ligand concentrations is shown by box plots at each time point. Each box has three lines, which from low to high indicate the lower quartile, median, and upper quartile ligand concentration values ([L]) of bacterium population at a given time. Whiskers extend from the box out to the most extreme data value within 1.5 folds of the height of the box. [L] values beyond whiskers are marked by points. Solid line shows the average of [L] against time.
(2.33 MB TIF)

Figure S3 Effect of variations of ω_1_, ω_2_ on the final average ligand concentration. Each data point represents the movement of 100 bacteria in 1000 s and their average ligand concentration in the last 50 s are calculated as a measurement of chemotactic effects. The transfer function, and max amplification (A), shape and parameters of the concentration field, and initial point of bacteria, are shown in Table S1.
(2.35 MB TIF)

Figure S4 CheY-P level changes caused by a step-wise signal in the band-pass filter. The parameters of the pathway are the same as that in Figure 3B. Attractants are removed at t = 0. The plot of [CheY-P] clearly indicates that the [CheY-P] reaches a high level in a short time (response time τ_2_ = 0.16 s) and recovers to the basal level gradually (adaptation time τ_1_ = 40 s).
(0.73 MB TIF)

Figure S5 Designed dynamics of positive, negative and pseudo- chemotaxis pathway and their block diagrams. (A) Positive chemotaxis pathway. Ligand quickly binds to molecule u to activate it. Activated u can dephosphorylate CheY-P and activate molecule v. Molecule v phosphorylates CheY-P. (B) Negative chemotaxis pathway. Ligand quickly binds to molecule u to activate it. Activated u can phosphorylate CheY-P and activate molecule v. Molecule v dephosphorylates CheY-P. (C) Pseudochemotaxis pathway. Ligand quickly binds to molecule u to activate it. Activated u can activate molecule v. Molecule v dephosphorylates CheY-P.
(0.57 MB TIF)

Table S1 Parameters of each subfigure in Figure S3.
(0.04 MB DOC)
