# Supplementary material for: Reverse Engineering of Bacterial Chemotaxis Pathway via Frequency Domain Analysis
Source: PLoS One. 2010 Mar 9;5(3):e9182. doi: 10.1371/journal.pone.0009182 (PMC2834735; doi:10.1371/journal.pone.0009182)
Supplement: Text S1 — Supplemental materials. Exposition and derivation of transfer function of pathway near equilibrium and the model for molecular motor in E. coli. (0.52 MB DOC) [file pone.0009182.s001.doc]

**Subject Categories:** Biological Science --- Synthetic biology, Signal Transduction

**Supplemental Materials**

**Reverse engineering of bacterial chemotaxis pathway via frequency domain analysis**

**Junjie Luo1, Jun Wang2, Ting Martin Ma1, and Zhirong Sun1***

**1**Department of Biological Sciences and Biotechnology, Tsinghua University, Ministry of Education Key Laboratory of Bioinformatics, Beijing, P.R.China 100084

**2**Department of Computer Science, Tsinghua University, Beijing, P.R.China 100084

****Correspondent author:***

Zhirong Sun, Department of Biological Sciences and Biotechnology, Tsinghua University, Ministry of Education Key Laboratory of Bioinformatics, Room 216, New Biological Building, Tsinghua University, Beijing, P.R. China 100084

E-mail: [sunzhr@mail.tsinghua.edu.cn](mailto:sunzhr@mail.tsinghua.edu.cn)

Tel: +86-10-62772237

Fax: +86-10-62772237

Transfer Function of Pathway near Equilibrium

The ordinary differential equation model for a signaling pathway can be written in following standard form:

$$\frac{d\vec{x}}{dt}=\vec{F}(\vec{x},\vec{u});\vec{y}=\vec{G}(\vec{x},\vec{u});$$

$\vec{u}$ is the input of this pathway, which can be the concentration of ligand or activated receptor. $\vec{x}$ is the molecular concentration in this pathway. $\vec{y}$ is pathway output, represents concentration of CheY-P in our research.

Set $\vec{u}_{0},\vec{x}_{0},\vec{y}_{0}$ are the equilibrium values of $\vec{u},\vec{x},\vec{y}$. So the three values obey equation group.

$$0=\vec{F}(\vec{x}_{0},\vec{u}_{0});\vec{y}_{0}=\vec{G}(\vec{x}_{0},\vec{u}_{0});$$

One order Taylor expansion of equation group is:

$$\left\{\begin{matrix} \frac{d(\Delta\vec{x}+\vec{x}_{0})}{dt}=\vec{F}(\vec{x}_{0},\vec{u}_{0})+\sum_{j} \left(\left.\frac{\partial\vec{F}}{\partial x_{j}}\right|_{\begin{matrix} \vec{x}=\vec{x}_{0}, \\ \vec{u}=\vec{u}_{0} \end{matrix}}\Delta x_{j}\right)+\sum_{j} \left(\left.\frac{\partial\vec{F}}{\partial u_{j}}\right|_{\begin{matrix} \vec{x}=\vec{x}_{0}, \\ \vec{u}=\vec{u}_{0} \end{matrix}}\Delta u_{j}\right)+o(\Delta\vec{x})+o(\Delta\vec{u}); \\ \Delta\vec{y}+\vec{y}_{0}=\vec{G}(\vec{x}_{0},\vec{u}_{0})+\sum_{j} \left(\left.\frac{\partial\vec{G}}{\partial x_{j}}\right|_{\begin{matrix} \vec{x}=\vec{x}_{0}, \\ \vec{u}=\vec{u}_{0} \end{matrix}}\Delta x_{j}\right)+\sum_{j} \left(\left.\frac{\partial\vec{G}}{\partial u_{j}}\right|_{\begin{matrix} \vec{x}=\vec{x}_{0}, \\ \vec{u}=\vec{u}_{0} \end{matrix}}\Delta u_{j}\right)+o(\Delta\vec{x})+o(\Delta\vec{u}); \end{matrix}\right.$$

Where $\Delta\vec{u}=\vec{u}-\vec{u}_{0},\Delta\vec{x}=\vec{x}-\vec{x}_{0},\Delta\vec{y}=\vec{y}-\vec{y}_{0}.$

Use equation group to simplify above equation group and ignore high order term of it, we can get following linear equation group:

$$\frac{d}{dt}\Delta\vec{x}=A\Delta\vec{x}+B\Delta\vec{u};\Delta\vec{y}=C\Delta\vec{x}+D\Delta\vec{u}$$

In which, $A_{ij}=\left.\frac{\partial F_{i}}{\partial x_{j}}\right|_{\vec{x}=\vec{x}_{0},\vec{u}=\vec{u}_{0}};B_{ij}=\left.\frac{\partial F_{i}}{\partial u_{j}}\right|_{\vec{x}=\vec{x}_{0},\vec{u}=\vec{u}_{0}};C_{ij}=\left.\frac{\partial G_{i}}{\partial x_{j}}\right|_{\vec{x}=\vec{x}_{0},\vec{u}=\vec{u}_{0}};D_{ij}=\left.\frac{\partial G_{i}}{\partial u_{j}}\right|_{\vec{x}=\vec{x}_{0},\vec{u}=\vec{u}_{0}}$

We will get the following formula from the Laplace transformation of :

$$s\Delta\vec{x}=A\Delta\vec{x}+B\Delta\vec{u};\Delta\vec{y}=C\Delta\vec{x}+D\Delta\vec{u}$$

Transfer function describing the dynamic relationship between $\Delta\vec{u}$ and $\Delta\vec{y}$ can be calculated from these equations by following formula:

$$H(s)=C(sI-A)^{-1}B+D$$

In our research, we use MATLAB function “*fsolve*” to solve the equilibrium equation group . This function also return the Jacobi matrix at equilibrium, which just correspond A, B, C, D in . This method enables us to get the transfer function directly from the standardized differential equation model shown in .

The ordinary differential of equation group is:

$$\begin{matrix} 0=\sum_{j} \left(\left.\frac{\partial F_{i}}{\partial x_{j}}\right|_{\begin{matrix} \vec{x}=\vec{x}_{0}, \\ \vec{u}=\vec{u}_{0} \end{matrix}}dx_{j0}\right)+\sum_{j} \left(\left.\frac{\partial F_{i}}{\partial u_{j}}\right|_{\begin{matrix} \vec{x}=\vec{x}_{0}, \\ \vec{u}=\vec{u}_{0} \end{matrix}}du_{j0}\right); \\ dy_{i0}=\sum_{j} \left(\left.\frac{\partial G_{i}}{\partial x_{j}}\right|_{\begin{matrix} \vec{x}=\vec{x}_{0}, \\ \vec{u}=\vec{u}_{0} \end{matrix}}dx_{j0}\right)+\sum_{j} \left(\left.\frac{\partial G_{i}}{\partial u_{j}}\right|_{\begin{matrix} \vec{x}=\vec{x}_{0}, \\ \vec{u}=\vec{u}_{0} \end{matrix}}du_{j0}\right); \end{matrix}$$

That is:

$$\begin{matrix} 0=Ad\vec{x}_{0}+Bd\vec{u}_{0} \\ d\vec{y}_{0}=Cd\vec{x}_{0}+Dd\vec{u}_{0} \end{matrix}$$

Eliminate $d\vec{x}_{0}$ in above equation group:

$$d\vec{y}_{0}=(-CA^{-1}B+D)d\vec{u}_{0}$$

If we solve equation group , we can get y0 as a function of u0. The partial differential of this function is:

$$\frac{\partial}{\partial u_{j0}}y_{i0}(\vec{u}_{0})=\left(-CA^{-1}B+D\right)_{ij}=H_{ij}(0)$$

Therefore, yi0 is not relative to uj0 is equivalent to Hij(0)=0. This provides a litmus stone to test whether a pathway is adaptive.

Systematic Design a Pathway Basing on Defined Transfer Function

The transfer function of a two-molecule pathway

Suppose a pathway is only made up of two molecules: receptor and CheY, whose concentrations are u and y. The dynamic of it can be described by following differential equation:

$$\frac{dy}{dt}=(C_{11}+C_{1}u)(y_{t}-y)-(D_{11}+D_{1}u)y$$

Let Eij=Cij+Dij. The above equation can be simplified into:

$$\frac{dy}{dt}=(C_{11}+C_{1}u)y_{t}-(E_{11}+E_{1}u)y$$

The equilibrium of this equation is:

$$y_{0}=\frac{C_{11}+C_{1}u_{0}}{E_{11}+E_{1}u_{0}}y_{t}$$

Calculation Taylor Series and Laplace Transformation near the equilibrium, the differential equation is approximated into following linear form:

$$s\Delta y=C_{1}\Delta uy_{t}-E_{1}\Delta uy_{0}-(E_{11}+E_{1}u_{0})\Delta y$$

Where Δu=u-u0; Δy=y-y0.

Therefore, the transfer function of the pathway is:

$$H(s)=\frac{\Delta y}{\Delta u}=\frac{C_{1}y_{t}-E_{1}y_{0}}{s+(E_{11}+E_{1}u_{0})}$$

This transfer function is one rank, thus the pathway it represents can only be a low-pass filter with cut-off frequency E11+E1u0. And this pathway is not adaptive.

The transfer function of three-molecule pathway

Suppose a pathway is made up of molecule u, v and CheY. The dynamic of it can be described by following differential equation group:

$$\left\{\begin{matrix} \frac{dv}{dt}=(C_{11}+C_{12}y+C_{1}u)(v_{t}-v)-(D_{11}+D_{12}y+D_{1}u)v \\ \frac{dy}{dt}=(C_{21}v+C_{22}+C_{2}u)(y_{t}-y)-(D_{21}v+D_{22}+D_{2}u)y \end{matrix}\right.$$

Let Eij=Cij+Dij. The above equation group can be simplified into:

$$\left\{\begin{matrix} \frac{dv}{dt}=(C_{11}+C_{12}y+C_{1}u)v_{t}-(E_{11}+E_{12}y+E_{1}u)v \\ \frac{dy}{dt}=(C_{21}v+C_{22}+C_{2}u)y_{t}-(E_{21}v+E_{22}+E_{2}u)y \end{matrix}\right.$$

Calculation Taylor Series and Laplace Transformation near the equilibrium (u0, v0, y0), the differential equation group is approximated into following linear form:

$$\left\{\begin{matrix} s\Delta v=(C_{12}\Delta y+C_{1}\Delta u)v_{t}-(E_{12}\Delta y+E_{1}\Delta u)v_{0}-(E_{11}+E_{12}y_{0}+E_{1}u_{0})\Delta v \\ s\Delta y=(C_{21}\Delta v+C_{2}\Delta u)y_{t}-(E_{21}\Delta v+E_{2}\Delta u)y_{0}-(E_{21}v_{0}+E_{22}+E_{2}u_{0})\Delta y \end{matrix}\right.$$

Where Δu=u-u0; Δv=v-v0; Δy=y-y0.

Substitute H(s)Δu for Δy, and eliminate Δu and Δv in :

$$\frac{s+(E_{11}+E_{12}y_{0}+E_{1}u_{0})}{-C_{21}y_{t}+E_{21}y_{0}}=\frac{(C_{12}H(s)+C_{1})v_{t}-(E_{12}H(s)+E_{1})v_{0}}{-sH(s)+C_{2}y_{t}-E_{2}y_{0}-(E_{21}v_{0}+E_{22}+E_{2}u_{0})H(s)}$$

Therefore, the transfer function of the pathway is:

$$H(s)=\frac{\left(s+(E_{11}+E_{12}y_{0}+E_{1}u_{0})\right)\left(C_{2}y_{t}-E_{2}y_{0}\right)-\left(-C_{21}y_{t}+E_{21}y_{0}\right)\left(C_{1}v_{t}-E_{1}v_{0}\right)}{\left(s+(E_{11}+E_{12}y_{0}+E_{1}u_{0})\right)\left(s+(E_{21}v_{0}+E_{22}+E_{2}u_{0})\right)+\left(-C_{21}y_{t}+E_{21}y_{0}\right)\left(C_{12}v_{t}-E_{12}v_{0}\right)}$$

This transfer function is two-rank, thus the pathway it represents can be a band-pass filter or a low-pass filter.

Let C1vt=k1, E11=dv, C21=E21=k3, E2=k2, and other Cij and Eij equal to 0. The equilibrium and transfer function can be simplified into:

$$\left\{\begin{matrix} y_{0}=y_{t}/\left(1+\frac{d_{v}}{k_{1}}\frac{k_{2}}{k_{3}}\right) \\ H(s)=\frac{\Delta y}{\Delta u}=\frac{-\left(k_{2}y_{t}/\left(1+d_{v}k_{2}/k_{1}k_{3}\right)\right)s}{\left(s+d_{v}\right)\left(s+u_{0}\left(k_{2}+k_{3}k_{1}/d_{v}\right)\right)} \end{matrix}\right.$$

This is the positive chemotaxis pathway we design.

Similarly, we can design a negative pathway by letting C1vt=k1, E11=dv, C2=E2=k2, E21=k3, and other Cij and Eij equal to 0:

$$\left\{\begin{matrix} y_{0}=y_{t}/\left(1+\frac{k_{1}}{d_{v}}\frac{k_{3}}{k_{2}}\right) \\ H(s)=\frac{\Delta y}{\Delta u}=\frac{\left(k_{2}y_{t}/\left(1+d_{v}k_{2}/k_{1}k_{3}\right)\right)s}{\left(s+d_{v}\right)\left(s+u_{0}\left(k_{2}+k_{3}k_{1}/d_{v}\right)\right)} \end{matrix}\right.$$

And a model of pseudochemotaxis pathway, by letting C1vt=k1, E11=dv, C22=E22=k2, E21=k3, and other Cij and Eij equal to 0:

$$\left\{\begin{matrix} y_{0}=y_{t}/\left(1+\frac{k_{1}}{d_{v}}\frac{k_{3}}{k_{2}}u_{0}\right) \\ H(s)=\frac{\Delta y}{\Delta u}=\frac{-y_{t}/\left(1/k_{3}k_{1}+u_{0}/d_{v}k_{2}\right)}{\left(s+d_{v}\right)\left(s+\left(k_{2}+u_{0}k_{3}k_{1}/d_{v}\right)\right)} \end{matrix}\right.$$

In our simulation, k1=dv=0.02s-1, k2=k3=20μM-1s-1(in , k2=10.9s-1), so the chemotaxis effects of the three pathway are comparable. And in , dv and (k2+u0k3k1/dv), the two polar points of the transfer function correspond to the speed of CheZ mRNA degradation and CheY-P dephosphorylation in the pseudochemotaxis pathway. For this reason, this model can simulate the pathway build by Topp S and Gallivan JP (Topp & Gallivan, 2007).

Transfer function of a pathway with discretionary number of molecules

Similarly, the differential equation describing the dynamic of the pathway can be written in following form (Goldstein & Soyer, 2008):

$$\frac{dy_{i}}{dt}=(C_{ii}+\sum_{j\neq i} C_{ij}y_{j}+C_{i}u)(y_{it}-y_{i})-(D_{ii}+\sum_{j\neq i} D_{ij}y_{j}+D_{i}u)y_{i}$$

Where u is the receptor occupation, yti is the total concentration of other proteins in the pathway except the receptor, and yi is the concentration of activated protein i. Cij (Dij) is the rate at which activated protein j activates (deactivates) protein i, Cii (Dii) is protein i’s rate of self-activation (deactivation), Ci (Di) represents the rate at which the receptor activates (deactivates) the protein i.

Let Eij=Cij+Dij. The above equation group can be simplified into:

$$\frac{dy_{i}}{dt}=(C_{ii}+\sum_{j\neq i} C_{ij}y_{j}+C_{i}u)y_{it}-(E_{ii}+\sum_{j\neq i} E_{ij}y_{j}+E_{i}u)y_{i}$$

Calculation Taylor Series and Laplace Transformation near the equilibrium (u0, v0, y0), the differential equation group is approximated into following linear form:

$$s\Delta y_{i}=\sum_{j\neq i} (C_{ij}y_{it}-E_{ij}y_{i0})\Delta y_{j}-(E_{ii}+\sum_{j\neq i} E_{ij}y_{j0}+E_{i}u_{0})\Delta y_{i}+(C_{i}y_{it}-E_{i}y_{i0})\Delta u$$

Where Δu=u-u0; Δyi=yi-yi0.

Let Aij=Cijyit-Eijyi0, (i≠j); Aii=-(Eii+∑k≠iEikyk0+Eiu0); Bi1=Ciyit-E­iyi0; ΔY=(y1,y2,…,yn)T.

$$s\Delta Y=A\Delta Y+B\Delta u$$

Therefore, the transfer function of the pathway is:

$$\begin{matrix} H(s)=(0,\cdots,0,1)\Delta Y/\Delta u=(0,\cdots,0,1)(sI-A)^{-1}B \\ =\frac{\det\left(\begin{matrix} s-A_{11} & -A_{12} & \cdots& -A_{1,n-1} & -A_{1n} \\ -A_{21} & \ddots& \ddots& \vdots& \vdots\\ \vdots& \ddots& \ddots& -A_{n-2,n-1} & \vdots\\ -A_{n-1,1} & \cdots& -A_{n-1,n-2} & s-A_{n-1,n-1} & -A_{n-1,n} \\ B_{11} & \cdots& \cdots& \cdots& B_{n1} \end{matrix}\right)}{\det\left(\begin{matrix} s-A_{11} & -A_{12} & \cdots& -A_{1n} \\ -A_{21} & \ddots& \ddots& \vdots\\ \vdots& \ddots& \ddots& -A_{n-1,n} \\ -A_{n,1} & \cdots& -A_{n,n-1} & s-A_{nn} \end{matrix}\right)} \end{matrix}$$

The number of the molecules in the pathway is (n+1) (yi (i=1 to n), and u). Equation shows that the rank of the transfer function is n, and the highest power of s in numerator is (n-1). Consequently, if we want to design an adaptive pathway and make the transfer function of it contain a differential link, n should be 2 at least. That is the smallest pathway fulfilling above properties has 3 molecules.

The Model for Molecular Motor in *E. coli*

We assume the change of molecular motor in *E. coli* is a Markov process (Block et al, 1983). That is the probability of motor rotation state at a time point is determined by the probability at the time point before. In following derivation, we use 0 to represent clockwise (CW) rotation and 1 to represent count-clockwise (CCW) rotation. The state transfer matrix of this Markov chain is:

$$P=\left(\begin{matrix} 1-p_{01} & p_{10} \\ p_{01} & 1-p_{10} \end{matrix}\right)$$

The *p01* stands for the probability of the motor changes from clockwise rotation to count-clockwise rotation. And *p10* stands for the probability of the motor changes from count-clockwise rotation to clockwise rotation.

In our simulation, the Markov process is assumed to be discrete, which means that the state of the motor does not change in a short time scale (Δt).

The research done by Cluzel P. et al measured the rotation bias (pCW) and switching frequency (fswitch) of the molecular motor under different CheY-P concentrations in single cell. We suppose [CheY-P] influence the behavior of the motor by changing the parameter in the state transfer matrix.

In this experiment, the time they monitoring the motor state is long enough, so we consider the probability of the motor on each state has become stable:

$$\left(\begin{matrix} p_{CW} \\ 1-p_{CW} \end{matrix}\right)=P\left(\begin{matrix} p_{CW} \\ 1-p_{CW} \end{matrix}\right)$$

This Equation can be simplified into following equation:

$$p_{120}=p_{CW}(p_{021}+p_{120})$$

Suppose a motor has changed its state n times in a time period kΔt, and its state is s (s=0 or 1). We use P(n,k,s) to represent the probability of this event. This probability follows equation group.

$$\left\{\begin{matrix} P(n,k,0)=(1-p_{01})P(n,k-1,0)+p_{10}P(n-1,k-1,1) \\ P(n,k,1)=(1-p_{10})P(n,k-1,1)+p_{01}P(n-1,k-1,0) \end{matrix}\right.$$

In above equation group, if n>k or n < 0 or k<0, P(n,k,s)=0.

Sum the two equations in, we can get:

$$\begin{matrix} (P(n,k,0)+P(n,k,1))-(P(n,k-1,0)+P(n,k-1,1)) \\ =p_{10}(P(n-1,k-1,1)-P(n,k-1,1))+p_{01}(P(n-1,k-1,0)-P(n,k-1,0)) \end{matrix}$$

$$\begin{matrix} \Rightarrow\\ \sum_{n=0}^{k} n(P(n,k,0)+P(n,k,1))-\sum_{n=0}^{k} n(P(n,k-1,0)+P(n,k-1,1)) \\ =p_{10}\sum_{n=0}^{k} n(P(n-1,k-1,1)-P(n,k-1,1))+p_{01}\sum_{n=0}^{k} n(P(n-1,k-1,0)-P(n,k-1,0)) \\ \Rightarrow\\ \sum_{n=0}^{k} n(P(n,k,0)+P(n,k,1))-\sum_{n=0}^{k} n(P(n,k-1,0)+P(n,k-1,1)) \\ =p_{10}\left(\sum_{n=0}^{k} P(n-1,k-1,1)+\sum_{n=0}^{k} ((n-1)P(n-1,k-1,1)-nP(n,k-1,1))\right) \\ +p_{01}\left(\sum_{n=0}^{k} P(n-1,k-1,0)+\sum_{n=0}^{k} ((n-1)P(n-1,k-1,0)-nP(n,k-1,0))\right) \\ \Rightarrow\end{matrix}$$

$$\begin{matrix} \sum_{n=0}^{k} n(P(n,k,0)+P(n,k,1))-\sum_{n=0}^{k} n(P(n,k-1,0)+P(n,k-1,1)) \\ =p_{10}\sum_{n=0}^{k} P(n-1,k-1,1)+p_{01}\sum_{n=0}^{k} P(n-1,k-1,0) \end{matrix}$$

Use the following 3 equations to eliminate P(n,k,s) in equation.

$$\begin{matrix} \sum_{n=0}^{k} n(P(n,k,0)+P(n,k,1))=f_{switch}k\Delta t; \\ \sum_{n=0}^{k} P(n,k,1)=1-p_{CW}; \\ \sum_{n=0}^{k} P(n,k,0)=p_{CW}; \end{matrix}$$

Then we can get the equation describing the correlation of fswitch, Δt, p10, p01, pCW:

$$f_{switch}\Delta t=p_{10}(1-p_{CW})+p_{01}p_{CW}$$

The p10, p01 in the state transfer matrix can be derived from equation and :

$$\left\{\begin{matrix} p_{01}=\frac{f_{switch}\Delta t}{2p_{CW}} \\ p_{10}=\frac{f_{switch}\Delta t}{2(1-p_{CW})} \end{matrix}\right.$$

The CW bias pCW­ and switching frequency fswitch as functions of CheY-P are cited from the research of Cluzel P. et al (Cluzel et al, 2000):

$$\left\{\begin{matrix} p_{CW}=\frac{([CheY-P]/K_{M})^{H}}{([CheY-P]/K_{M})^{H}+1}; \\ f_{switch}=f_{M}\frac{4([CheY-P]/K_{M})^{H-1}}{(([CheY-P]/K_{M})^{H}+1)^{2}}\propto\frac{\partial p_{CW}}{\partial[CheY-P]}; \end{matrix}\right.$$

Where Hill coefficient H=10.3, KM=3.1 μM, fM=1.7 Hz.


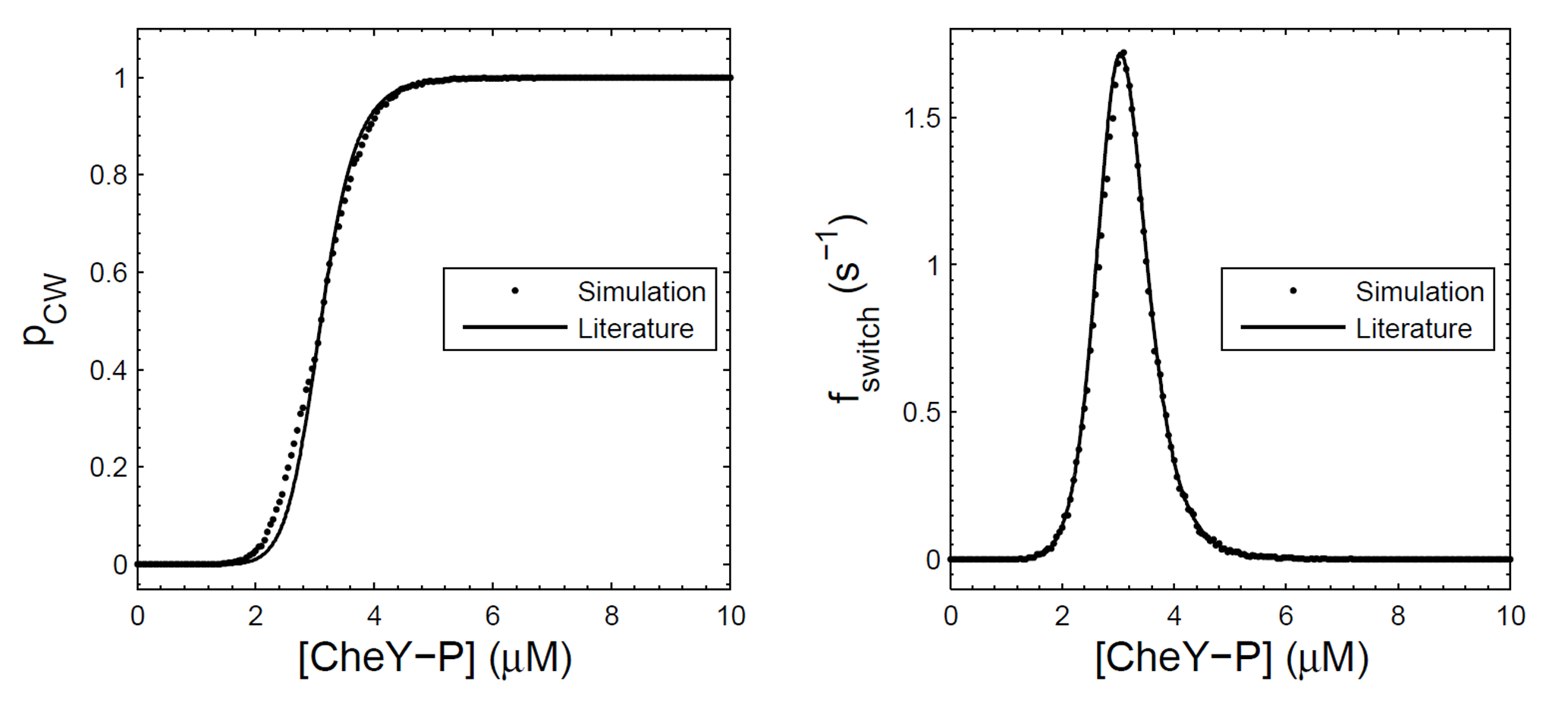


**Figure S6.** Characteristic response of motors against CheY-P concentrations. Each point describes a simulation of the motor under a certain CheY-P concentration. The states of motor in 2500s are recorded and CW bias and switching frequency are calculated from them. The solid lines are the curve of functions . The simulation results fit the curve from literature very well.

The probability density of bacteria with parameter x, y, D, s: $f(\vec{x},\vec{y},\vec{D},s,t)$

$\vec{x}$: position of bacteria; $\vec{y}$: concentration of molecules related to chemotaxis in bacteria; $\vec{D}$: direction vector of bacteria; $s$: state of motor on bacteria; t: time.

Suppose the change of the molecules in chemotaxis pathway can be represented by following standard differential equations:

$$\frac{\partial\vec{y}}{\partial t}=\vec{F}(\vec{y},L(\vec{x}));$$

This equation group contains the information about concentration field and the interaction of molecules in cell.

Let the average speed of the bacteria is v, we can get the derivation of x:

$$\frac{\partial\vec{x}}{\partial t}=v\vec{D};$$

In our simulation, we suppose during a small time scale Δt, the state of a bacterium changes from $(\vec{x}',\vec{y}',\vec{D}',s',t)$ to $(\vec{x},\vec{y},\vec{D},s',t)$, and at the end of this time scale, the bacterium’s state switches into $(\vec{x},\vec{y},\vec{D},s,t)$

If the motor state s'=0:

$$\begin{matrix} \vec{x}'=\vec{x};\vec{y}'=\vec{y}-\Delta\vec{y}=\vec{y}-\frac{\partial\vec{y}}{\partial t}\Delta t=\vec{y}-\vec{F}\Delta t;\vec{D}'=\vec{D}-\Delta\vec{D} \\ d\vec{x}'d\vec{y}'d\vec{D}'=\left|\frac{D(\vec{x}',\vec{y}',\vec{D}')}{D(\vec{x},\vec{y},\vec{D})}\right|d\vec{x}d\vec{y}d\vec{D}=\left|\begin{matrix} I & 0 & 0 \\ -\frac{\partial\vec{F}}{\partial\vec{x}}\Delta t & I-\frac{\partial\vec{F}}{\partial\vec{y}}\Delta t & 0 \\ 0 & 0 & I \end{matrix}\right|d\vec{x}d\vec{y}d\vec{D}=\left|I-\frac{\partial\vec{F}}{\partial\vec{y}}\Delta t\right|d\vec{x}d\vec{y}d\vec{D} \end{matrix}$$

If the motor state s'=1:

$$\begin{matrix} \vec{x}'=\vec{x}-\Delta\vec{x}=\vec{x}-\vec{D}v\Delta t;\vec{y}'=\vec{y}-\Delta\vec{y}=\vec{y}-\frac{\partial\vec{y}}{\partial t}\Delta t=\vec{y}-\vec{F}\Delta t;\vec{D}'=\vec{D} \\ d\vec{x}'d\vec{y}'d\vec{D}'=\left|\frac{D(\vec{x}',\vec{y}',\vec{D}')}{D(\vec{x},\vec{y},\vec{D})}\right|d\vec{x}d\vec{y}d\vec{D}=\left|\begin{matrix} I & 0 & Iv\Delta t \\ -\frac{\partial\vec{F}}{\partial\vec{x}}\Delta t & I-\frac{\partial\vec{F}}{\partial\vec{y}}\Delta t & 0 \\ 0 & 0 & I \end{matrix}\right|d\vec{x}d\vec{y}d\vec{D}=\left|I-\frac{\partial\vec{F}}{\partial\vec{y}}\Delta t\right|d\vec{x}d\vec{y}d\vec{D} \end{matrix}$$

Then we can get a equation group describing the change of the probability density of bacteria parameters in Δt:

$$\left(\begin{matrix} f(\vec{x},\vec{y},\vec{D},0,t+\Delta t)d\vec{x}d\vec{y}d\vec{D} \\ f(\vec{x},\vec{y},\vec{D},1,t+\Delta t)d\vec{x}d\vec{y}d\vec{D} \end{matrix}\right)=\left(\begin{matrix} 1-p_{01}(\vec{y}) & p_{10}(\vec{y}) \\ p_{01}(\vec{y}) & 1-p_{10}(\vec{y}) \end{matrix}\right)\left(\begin{matrix} \iint dP(\Delta\vec{D})f(\vec{x},\vec{y}-\Delta\vec{y},\vec{D}-\Delta\vec{D},0,t)\left|I-\frac{\partial\vec{F}}{\partial\vec{y}}\Delta t\right|d\vec{x}d\vec{y}d\vec{D} \\ f(\vec{x}-\Delta\vec{x},\vec{y}-\Delta\vec{y},\vec{D},1,t)\left|I-\frac{\partial\vec{F}}{\partial\vec{y}}\Delta t\right|d\vec{x}d\vec{y}d\vec{D} \end{matrix}\right)$$

The CW bias and switching frequency of the motor can be measured in experiment, let they are the function of [CheY-P]: fswitch(y­n), PCW(yn). As mentioned above:

$$k_{01}=\frac{f_{switch}(y_{n})}{2p_{CW}(y_{n})};k_{10}=\frac{f_{switch}(y_{n})}{2(1-p_{CW}(y_{n}))};$$

The differential coefficient of some parameters can be derived from above equation group:

$\frac{\partial}{\partial t}\left\langle L(\vec{x})\right\rangle=\left\langle sv\sum_{i} D_{i}\frac{\partial L(\vec{x})}{\partial x_{i}}\right\rangle$; $\frac{\partial}{\partial t}\left\langle x_{i}\right\rangle=\left\langle svD_{i}\right\rangle$; $\frac{\partial}{\partial t}\left\langle y_{i}\right\rangle=\left\langle F_{i}(\vec{y},L(\vec{x}))\right\rangle$; $\frac{\partial}{\partial t}\left\langle D_{i}\right\rangle=\frac{E\left(\Delta D_{i}\right)}{\Delta t}\left\langle1-s\right\rangle$

These equations consist to the dynamics relations of a single bacterium and our simulation results.

If we simulate the movement of bacteria in 2D space, their moving direction can be represented by the angle θ, and Δθ can be considered as a random walk process:

$$\begin{matrix} E\left(\Delta\theta\right)=0;D\left(\Delta\theta\right)=k\Delta t; \\ \frac{\partial\vec{x}}{\partial t}=v\left(\begin{matrix} \cos\theta\\ \sin\theta\end{matrix}\right); \end{matrix}$$

Use testing the chemotaxis effects of the band-pass filter as an example:

$$\frac{\partial\vec{y}}{\partial t}=\left(\begin{matrix} 0 & -\omega_{1}\omega_{2} \\ 1 & -(\omega_{1}+\omega_{2}) \end{matrix}\right)\left(\begin{matrix} y_{1} \\ y_{2}-y_{0} \end{matrix}\right)+\left(\begin{matrix} 0 \\ -A(\omega_{1}+\omega_{2}) \end{matrix}\right)\frac{L}{L+K_{L}};L=L_{0}\exp\left(-\left(\frac{x_{1}}{r}\right)^{2}\right);$$

Consequently, the total equation describing the change of the distribution of bacteria population is:

$$\begin{matrix} \frac{\partial}{\partial t}\left(\begin{matrix} f(x,\vec{y},\theta,0,t) \\ f(x,\vec{y},\theta,1,t) \end{matrix}\right)+\left(\begin{matrix} -\frac{k}{2}\frac{\partial^{2}}{\partial\theta^{2}}f(\vec{x},\vec{y},\theta,0,t) \\ \left(v\cos\theta\frac{\partial}{\partial x_{1}}+v\sin\theta\frac{\partial}{\partial x_{2}}\right)f(\vec{x},\vec{y},\theta,1,t) \end{matrix}\right)+\frac{\partial}{\partial y_{1}}\left(-\omega_{1}\omega_{2}(y_{2}-y_{0})\left(\begin{matrix} f(x,\vec{y},\theta,0,t) \\ f(x,\vec{y},\theta,1,t) \end{matrix}\right)\right)+\frac{\partial}{\partial y_{2}}\left(\left(y_{1}-(\omega_{1}+\omega_{2})(y_{2}-y_{0}+\frac{A}{1+\frac{K_{L}}{L_{0}}\exp\left(\left(\frac{x_{1}}{r}\right)^{2}\right)})\right)\left(\begin{matrix} f(x,\vec{y},\theta,0,t) \\ f(x,\vec{y},\theta,1,t) \end{matrix}\right)\right) \\ =\frac{f_{switch}(y_{2})}{2}\left(\begin{matrix} 1 \\ -1 \end{matrix}\right)\left(\begin{matrix} -\frac{1}{p_{CW}(y_{2})} & \frac{1}{1-p_{CW}(y_{2})} \end{matrix}\right)\left(\begin{matrix} f(x,\vec{y},\theta,0,t) \\ f(x,\vec{y},\theta,1,t) \end{matrix}\right) \end{matrix}$$

Reference

Block SM, Segall JE, Berg HC (1983) Adaptation kinetics in bacterial chemotaxis. *J Bacteriol* **154:** 312-323

Cluzel P, Surette M, Leibler S (2000) An ultrasensitive bacterial motor revealed by monitoring signaling proteins in single cells. *Science* **287:** 1652-1655

Goldstein RA, Soyer OS (2008) Evolution of taxis responses in virtual bacteria: non-adaptive dynamics. *PLoS Comput Biol* **4:** e1000084

Topp S, Gallivan JP (2007) Guiding bacteria with small molecules and RNA. *J Am Chem Soc* **129:** 6807-6811
