# Supplementary figures and images for: Reverse Engineering of Bacterial Chemotaxis Pathway via Frequency Domain Analysis
Source: PLoS One. 2010 Mar 9;5(3):e9182. doi: 10.1371/journal.pone.0009182 (PMC2834735; doi:10.1371/journal.pone.0009182)

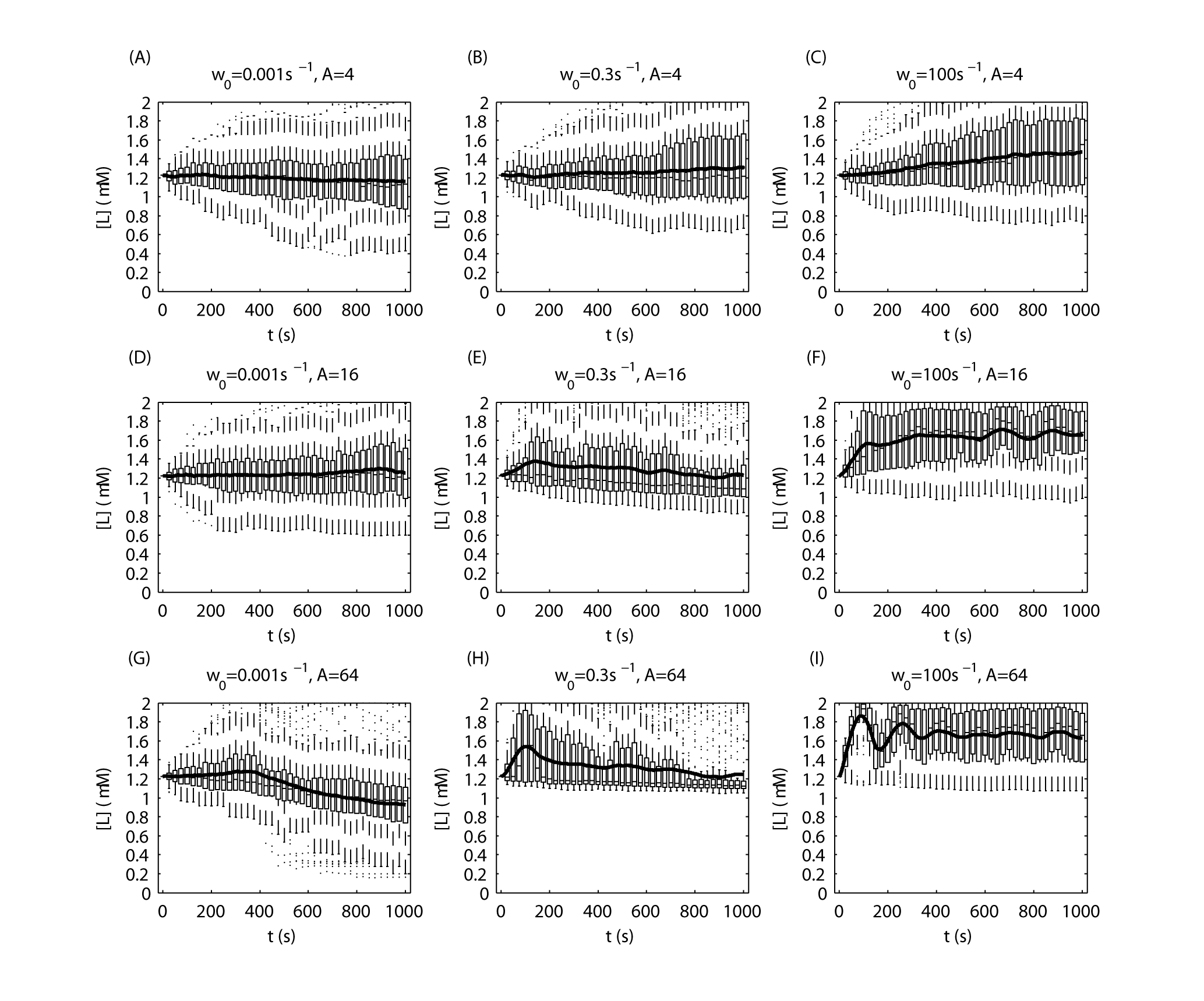

Supplement: Figure S1 — Chemotaxis behaviors of cells guided by low-pass filter with various parameters (ω0, A, whose values are shown above each subfigure) in a mountain-shaped concentration field. The concentration field is mountain-shaped (L = L0exp(−x2/r2), L0 = 2 µM, r = 2 mm). In each subfigure, 100 bacteria start at (1.4 mm, 0 mm) and their movement in 1000s is recorded. The distribution of their local ligand concentrations is shown by box plots at each time point. Each box has three lines, which from low to high indicate the lower quartile, median, and upper quartile ligand concentration values ([L]) of bacterium population at a given time. Whiskers extend from the box out to the most extreme data value within 1.5 folds of the height of the box. [L] values beyond whiskers are marked by points. Solid line shows the average of [L] against time. (2.11 MB TIF) [file pone.0009182.s002.tif]

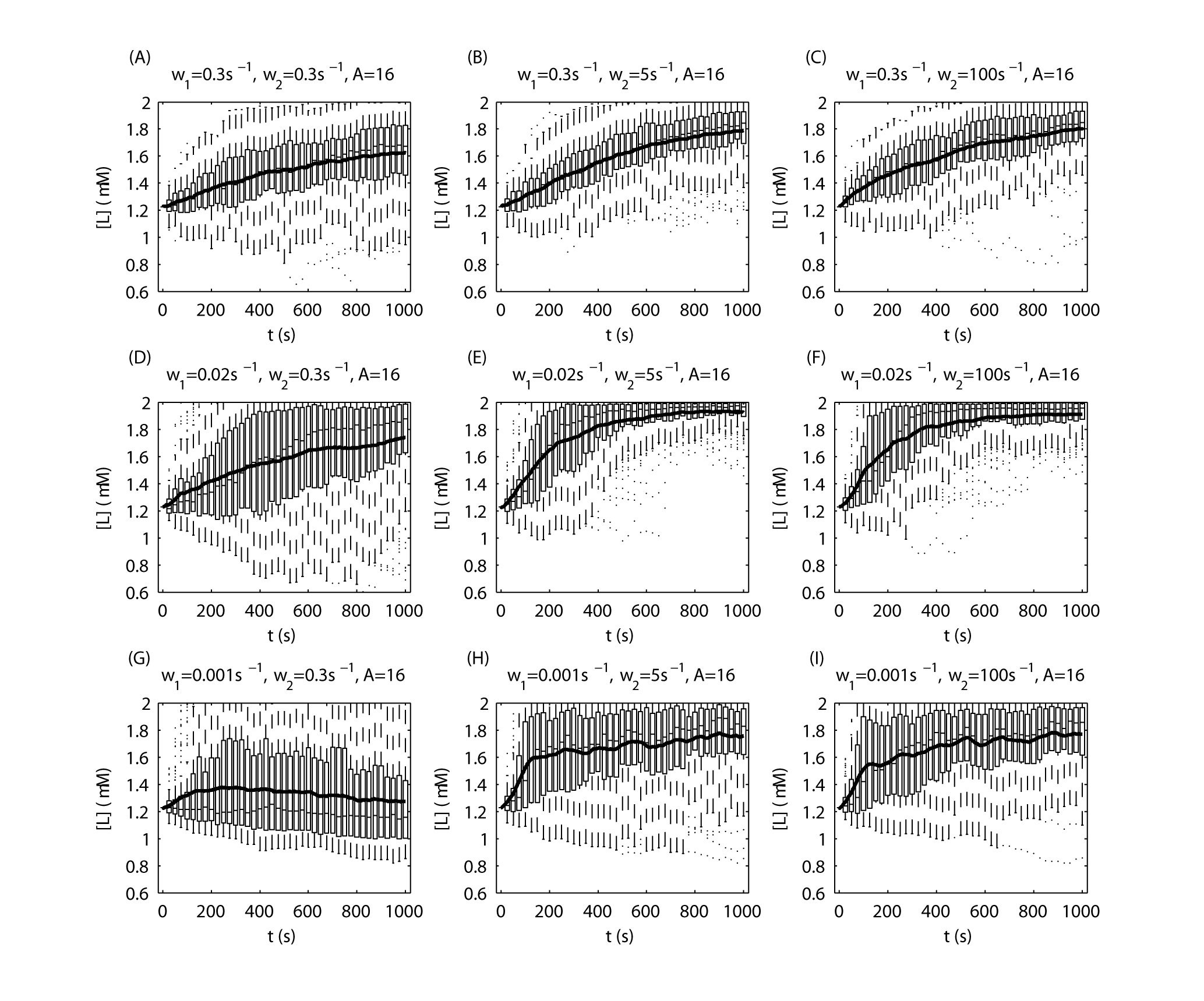

Supplement: Figure S2 — Chemotaxis behaviors of cells guided by band-pass filter with various cut-off frequencies (ω1, ω2, whose values are shown above each subfigure) in a mountain-shaped concentration field. The max amplification of the filter is fixed at 16. The concentration field is mountain-shaped (L = L0exp(−x2/r2), L0 = 2 µM, r = 2 mm). In each subfigure, 100 bacteria start at (1.4 mm, 0 mm) and their movement in 1000s is recorded. The distribution of their local ligand concentrations is shown by box plots at each time point. Each box has three lines, which from low to high indicate the lower quartile, median, and upper quartile ligand concentration values ([L]) of bacterium population at a given time. Whiskers extend from the box out to the most extreme data value within 1.5 folds of the height of the box. [L] values beyond whiskers are marked by points. Solid line shows the average of [L] against time. (2.33 MB TIF) [file pone.0009182.s003.tif]

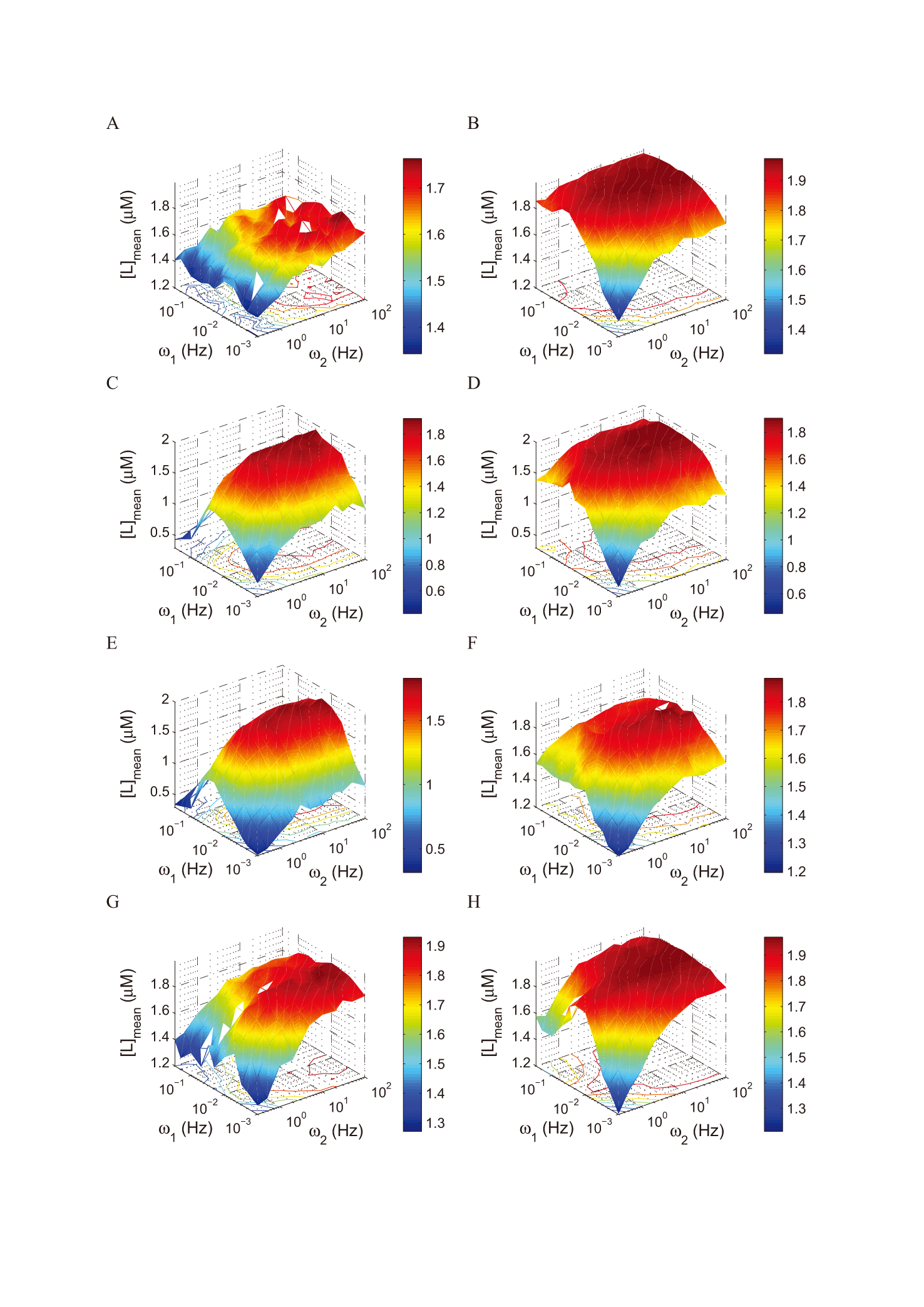

Supplement: Figure S3 — Effect of variations of ω1, ω2 on the final average ligand concentration. Each data point represents the movement of 100 bacteria in 1000 s and their average ligand concentration in the last 50 s are calculated as a measurement of chemotactic effects. The transfer function, and max amplification (A), shape and parameters of the concentration field, and initial point of bacteria, are shown in Table S1. (2.35 MB TIF) [file pone.0009182.s004.tif]

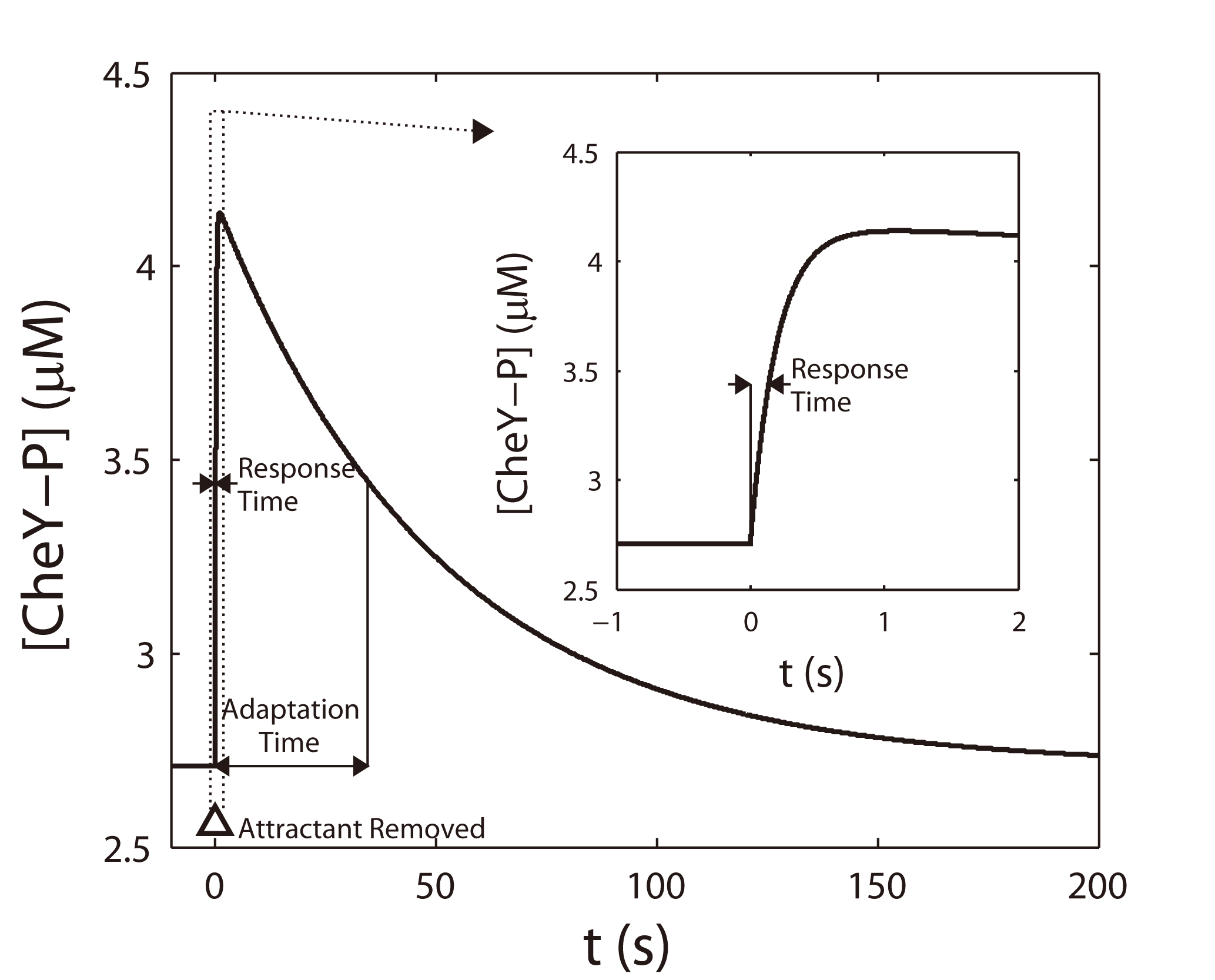

Supplement: Figure S4 — CheY-P level changes caused by a step-wise signal in the band-pass filter. The parameters of the pathway are the same as that in Figure 3B. Attractants are removed at t = 0. The plot of [CheY-P] clearly indicates that the [CheY-P] reaches a high level in a short time (response time τ2 = 0.16 s) and recovers to the basal level gradually (adaptation time τ1 = 40 s). (0.73 MB TIF) [file pone.0009182.s005.tif]

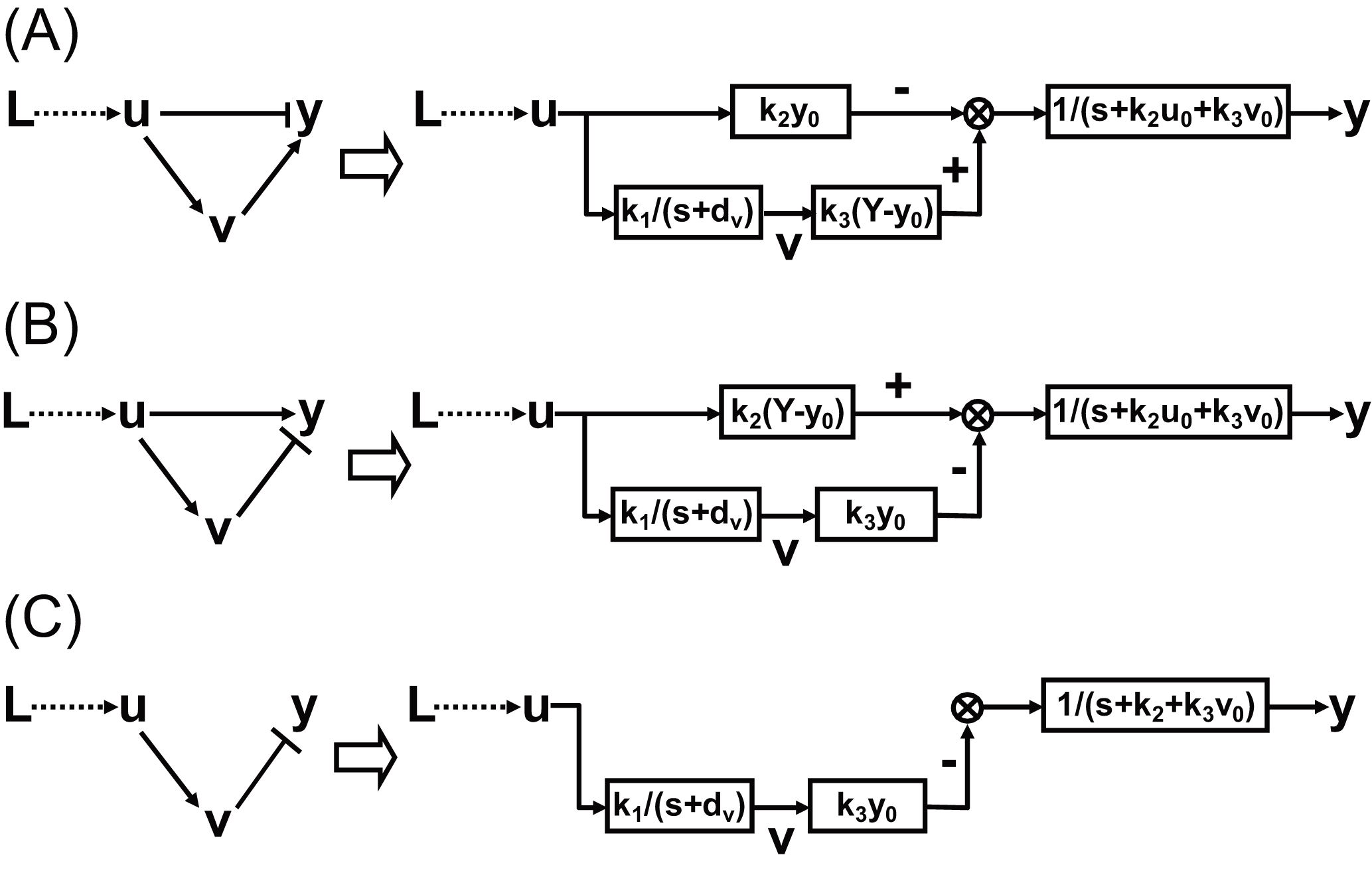

Supplement: Figure S5 — Designed dynamics of positive, negative and pseudo- chemotaxis pathway and their block diagrams. (A) Positive chemotaxis pathway. Ligand quickly binds to molecule u to activate it. Activated u can dephosphorylate CheY-P and activate molecule v. Molecule v phosphorylates CheY-P. (B) Negative chemotaxis pathway. Ligand quickly binds to molecule u to activate it. Activated u can phosphorylate CheY-P and activate molecule v. Molecule v dephosphorylates CheY-P. (C) Pseudochemotaxis pathway. Ligand quickly binds to molecule u to activate it. Activated u can activate molecule v. Molecule v dephosphorylates CheY-P. (0.57 MB TIF) [file pone.0009182.s006.tif]
