# Supplementary material for: Reverse Engineering of Bacterial Chemotaxis Pathway via Frequency Domain Analysis
Source: PLoS One. 2010 Mar 9;5(3):e9182. doi: 10.1371/journal.pone.0009182 (PMC2834735; doi:10.1371/journal.pone.0009182)
Supplement: Table S1 — Parameters of each subfigure in Figure S3. (0.04 MB DOC) [file pone.0009182.s007.doc]

**Table S1.** Parameters of each subfigure in Figure S3

| Subfigure | Transfer function | A | Concentration field | Initial point |
| --- | --- | --- | --- | --- |
| A | -A(ω_1_+ω_2_)s  (s+ω_1_)(s+ω_2_) | 8 | L=L_0_exp(-x^2^/r^2^)  L_0_=2 μM, r=2 mm | (1.4 mm, 0 mm) |
| B | -A(ω_1_+ω_2_)s  (s+ω_1_)(s+ω_2_) | 32 | L=L_0_exp(-x^2^/r^2^)  L_0_=2 μM, r=2 mm | (1.4 mm, 0 mm) |
| C | -A(ω_1_+ω_2_)s  (s+ω_1_)(s+ω_2_) | 16 | L=L_0_exp(-x^2^/r^2^)  L_0_=2 μM, r=2 mm | (3.5 mm, 0 mm) |
| D | -A(ω_1_+ω_2_)s  (s+ω_1_)(s+ω_2_) | 8 | L=L_0_exp(-x^2^/r^2^)  L_0_=2 μM, r=1 mm | (1.4 mm, 0 mm) |
| E | -A(ω_1_+ω_2_)s  (s+ω_1_)(s+ω_2_) | 16 | L=L_0_exp(-(x^2^+y^2^)/r^2^)  L_0_=2 μM, r=2 mm | (3.5 mm, 0 mm) |
| F | -A(ω_1_+ω_2_)s  (s+ω_1_)(s+ω_2_) | 16 | L=L_0_exp(-(x^2^+y^2^)/r^2^)  L_0_=2 μM, r=2 mm | (1.4 mm, 0 mm) |
| G | -A(ω_1_+ω_2_)s^2^  (s+ω_1_)^2^(s+ω_2_)^2^ | 16 | L=L_0_exp(-x^2^/r^2^)  L_0_=2 μM, r=2 mm | (1.4 mm, 0 mm) |
| H | -A(ω_1_+ω_2_)s^2^  (s+ω_1_)^2^(s+ω_2_)^2^ | 32 | L=L_0_exp(-x^2^/r^2^)  L_0_=2 μM, r=2 mm | (1.4 mm, 0 mm) |
